# Paradigm shifts and the interplay between state, business and civil sectors

**DOI:** 10.1098/rsos.160753

**Published:** 2016-12-21

**Authors:** Sara Encarnação, Fernando P. Santos, Francisco C. Santos, Vered Blass, Jorge M. Pacheco, Juval Portugali

**Affiliations:** 1 ESLab Environmental Simulation Laboratory, Tel Aviv University, Tel Aviv 69978, Israel; 2 ATP-group, 1649-003 Lisboa Codex, Portugal; 3 Interdisciplinary Centre of Social Sciences-CICS.NOVA-FCSH/UNL, Avenida de Berna, 26-C, 1069-061 Lisboa, Portugal; 4 INESC-ID and Instituto Superior Técnico, Universidade de Lisboa, IST-Taguspark, 2744-016 Porto Salvo, Portugal; 5 Coller School of Management, Tel Aviv University, Tel Aviv 69978, Israel; 6 Centro de Biologia Molecular e Ambiental and Departamento de Matemática e Aplicações, Universidade do Minho, 4710-057 Braga, Portugal

**Keywords:** multiple sectors, evolutionary dynamics, complexity, governance, policy

## Abstract

The recent rise of the civil sector as a main player of socio-political actions, next to public and private sectors, has largely increased the complexity underlying the interplay between different sectors of our society. From urban planning to global governance, analysis of these complex interactions requires new mathematical and computational approaches. Here, we develop a novel framework, grounded on evolutionary game theory, to envisage situations in which each of these sectors is confronted with the dilemma of deciding between maintaining a *status quo* scenario or shifting towards a new paradigm. We consider multisector conflicts regarding environmentally friendly policies as an example of application, but the framework developed here has a considerably broader scope. We show that the public sector is crucial in initiating the shift, and determine explicitly under which conditions the civil sector—reflecting the emergent reality of civil society organizations playing an active role in modern societies—may influence the decision-making processes accruing to other sectors, while fostering new routes towards a paradigm shift of the society as a whole. Our results are shown to be robust to a wide variety of assumptions and model parametrizations.

## 1. Introduction

The interrelationship among different sectors of society has evolved in the past four decades and became more intricate than before [1,2]. We have witnessed the emergence of a new ‘third sector’ [3] as a result of an increasingly organized civil society [4]—a process driven by grand socio-political and cultural processes such as globalization [5,6], technological innovation [1,7], privatization and the consequent weakening of the nation state [8,9]. As a result, the socio-economic and political process has been shifting from a two-player interaction (between public and private sectors) where the public sector takes care of some public goods, externalities and welfare activities, into a three-player interaction between public, private and civil sectors. This transformation entailed changes of known interdependencies: private and civil organizations often require state intervention through, e.g. (i) the establishment of rules [10] and/or (ii) the creation of new structures necessary to enable civil engagement [11], while also depending (some of them) on state policies, such as taxes and subsidies/incentives. Consequently, the political realm shifted from a regulatory state model to one often described as multi-level governance, network governance or self-governance [12]. The above-mentioned transformation led to a public sector that is no longer the only representative of society, given the role now played by the civil society sector. The recent dominant notion of ‘governance’ (over ‘government’) reflects this shift. These new dynamics are notorious, for instance, in the case of environmental protection and conservation, sustainable urban planning or diffusion of new environmental technologies, increasingly complex and costly enterprises that require effective cooperation between different actors at different levels [13,14]. As such, the set of instruments and policies proposed for dealing with sustainability issues, both at local and global levels, has grown in number and diversity [10,15–19].

Given the interdependencies between all three sectors (public, private and civil), and taking into account the conflicting interests that often characterize their interplay, it has proven hard to unveil the effectiveness of different social and/or political instruments in general. Indeed, these complex relationships—and ensuing social dynamics—are far easier to allude to than to quantitatively characterize. Interestingly, however, such complexities emerging from multiple populations and wide ranges of interdependencies also prevail in many ecologies, which have led to the integration of those nonlinearities inherent to complex adaptive systems into tractable models of ecology [20,21]. Our understanding of human socio-economic systems—from governance of global commons and finance, to urban planning and the adoption of public health measures—can only benefit from similar approaches [19,22–25], grounded, for instance, on the ecology of actions in populations of self-regarding parties [26–28].

Here, we model decision-making accruing to different sectors of society, identifying the core dynamics of conflict and cooperation underlying intersectorial interactions. To this end, we develop a simple yet novel modelling approach (see Methods), based on evolutionary game theory (EGT) [29–31], that involves three populations representing the aforementioned sectors of society. In each population, individuals have two options (also called strategies) concerning what to do regarding the adoption of a new paradigm (e.g. shifting into more sustainable societies). These two strategies, in all cases, can be modelled as being in favour (cooperation) and against (defection) the new paradigm. Our results show that, to depart from the *status quo*—associated with a minority of cooperators in each of the different sectors—the conjugated action of the private and civil sectors, alone, is not sufficient. On the other hand, the civil sector proves crucial in ensuring a sustainable scenario in which overall cooperation is achieved. In the following, we develop what we call the *core model*, containing the key features that generally accrue to these types of problems, leaving to the electronic supplementary material a specific implementation of the model designed to investigate a potential shift into a more sustainable society. The simpler, yet more abstract, core model allows us to introduce the underlying mathematical framework and the methods we employ in analysing the multi-population dynamics. Indeed, more sophisticated models, such as the one discussed in the electronic supplementary material, merely require consideration of a different payoff matrix.

## 2. Modelling framework

Let us consider three different populations of sizes *Z*1, *Z*2 and *Z*3. Besides realistic, the finite size of the populations is important, as, in general, one may expect the relative size of these three populations to be different. Each population represents one sector of society, namely the public, private and civil sectors. The public sector represents governmental institutions, the private sector represents companies that produce or sell products and the civil sector is composed by citizens that can manifest their preferences, either individually or by participating in e.g. non-governmental organizations (NGOs).

Each individual (in any of the three populations) can adopt one of two strategies: in favour of new (say, green, see electronic supplementary material) policies (cooperators, *C*s) and against (defectors, *D*s, in favour of maintaining the present *status quo*). The individual payoff earned in any one encounter depends on the strategy of the participating individuals. An encounter involves the participation of three individuals, each belonging to a different population. From this three-player game, each individual will acquire a payoff that reflects the mechanisms at stake, encoded in the payoff matrix detailed in table 1. This leads, in the general case, to a frequency-dependent dynamics in each of the three populations, where the dynamical process of strategy adoption assumes that those individuals that are more fit are more often imitated by their peers, an adaptive scheme akin to social learning [26,28,30,32]. Thus, contrary to what may be assumed in the realm of biological applications [33], the term ‘evolution’ in EGT simply refers to a variety of mechanisms that may lead to changes in behaviours or individual choices in time (see Methods for details).

**Table 1.** Payoff matrix, where *C* indicates a cooperator and *D* a defector. This minimal payoff structure covers the scenario in which (i) the civil sector may confer reputational benefits (*b*_R_) to the public sector whenever both sectors support the paradigm shift; (ii) private companies that embrace the paradigm shift entail a technological cost (*c*_T_), yet, doing so may entitle them to receive a subsidy benefit (*b*_S_) provided by the public sector at a cost *c*_S_; (iii) a civil sector opting for the paradigm shift may also benefit (for simplicity, we assume the same value *b*_S_) and (iv) whenever civil and private sectors are strategically aligned, a potential synergistic effect *σ* may occur.

strategies			payoffs accruing to each player		
public	private	civil	public	private	civil
*C*	*C*	*C*	*b*_R_ − 2*c*_S_	*σ* + *b*_S_ − *c*_T_	*σ* + *b*_S_
*C*	*C*	*D*	*−c*_S_	*b*_S_ − *c*_T_	0
*C*	*D*	*C*	*b*_R_ − *c*_S_	0	*b*_S_
*C*	*D*	*D*	0	*σ*	*σ*
*D*	*C*	*C*	0	*σ* − *c*_T_	*σ*
*D*	*C*	*D*	0	−*c*_T_	0
*D*	*D*	*C*	0	0	0
*D*	*D*	*D*	0	*σ*	*σ*

Table 1 shows the payoff matrix of the core model, in which we materialize the considerations above in a set of specific parameters. The rationale involved associates payoffs with variations with respect to the *status quo* situation: whenever the public sector cooperates (*C*), it may acquire a reputation benefit (*b*_R_ ≥ 0) to the extent that the civil sector is aligned with the same strategy. On the contrary, no such benefit will accrue when the civil sector defects (*D*). Moreover, a *C* public sector may be willing to subsidize (at a cost *c*_S_ ≥ 0) the (say, technological) means for the private and civil sectors to shift into the new paradigm [34], which will then result in a corresponding benefit (*b*_S_ ≥ 0); this may happen contingent on a strategic alignment between the public sector and each of the other two. Engaging in the new paradigm, in turn, entails a (technological) cost *c*_T_ (*c*_T_ ≥ 0) to the private sector. Finally, whenever the civil and private sectors are strategically aligned, there is a potential synergistic effect (*σ* ≥ 0) between the two [2], independent of the strategy of the public sector.

It is worth pointing out that when the paradigm shift is associated with the overall adoption of green policies (see electronic supplementary material), the complexity of the payoff matrix is augmented, because we take into consideration additional important effects, such as costly punishment [35,36] of a defecting public sector, and the possible punishment of a defecting private sector by a watchful and displeased civil sector. Such social punishment [37] mechanisms can induce considerable costs to both public and private sectors, as governments cannot easily ignore their electorate and firms can hardly afford negative publicity without greater costs to themselves. All these more sophisticated mechanisms go into the payoff matrix; nonetheless, the characterization of the overall dynamics proceeds in very much the same way as discussed in the following, in connection with the core model.

## 3. Results

**Figure 1.**
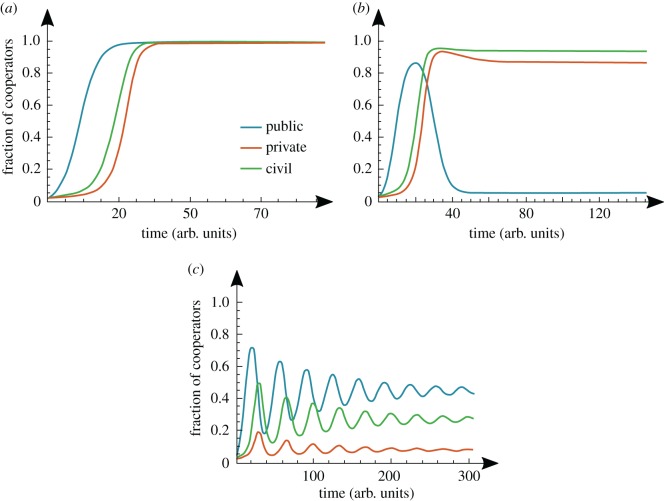
Evolutionary dynamics of a social paradigm shift. Starting from a *status quo* situation in which only 1% of individuals in each sector cooperate, as time unfolds, the public sector is able to initiate a transformation into cooperation in other sectors of society. We observe that the shift of the public sector is first followed by the civil sector and, finally, by the private sector. Depending on the relative magnitude of the model parameters, we may reach situations of full cooperation, reflecting a 100% successful paradigm shift (*a*); situations in which civil and private sectors shift into a cooperative configuration that becomes stable irrespective of the public sector (*b*) and also situations in which a (damped) cyclic behaviour is observed, in which the private sector is never drawn into cooperation (*c*). Model parameters: (*a*) *c*_S_ = *c*_T_ = 0.15, *b*_S_ = 0.4, *b*_R_ = 0.8, *σ* = 0.2; (*b*) *c*_S_ = *c*_T_ = 0.15, *b*_S_ = 0.4, *b*_R_ = 0, *σ* = 0.2; (*c*) *c*_T_ = 0.2, *c*_S_ = 0.5*, b*_S_ = 0.6, *b*_R_ = 0, *σ* = 0.3; other parameters, detailed in Methods: *β* = 2.5 *μ* = 0.02, *Z* = 50.

In figure 1, we anticipate the overall scenarios predicted by investigating the evolutionary dynamics of the core model. Details of the numerical simulations involved are provided in the Methods section. We start from a *status quo* state in which only a few cooperate (1% in each sector), following the subsequent time-series portraying the behavioural composition of each population. As shown in figure 1, a change in the public sector induces important consequences in the remaining sectors of society: in the first scenario (figure 1*a*), the civil sector is the first to follow the shift of the public sector into cooperation, the private sector being the last to shift. Once all sectors have shifted, the new paradigm becomes fully stable. In the scenario portrayed in figure 1*b*, a similar evolution takes place, except that in this case the subsidies costs are not compensated by the reputation feedback to the public sector provided by the civil sector. This, in turn, renders the cooperative scenario unstable for the public sector, yet without affecting the mutual stability between the private and civil sectors. Finally, in the third scenario illustrated in figure 1*c*, the complex interplay between the three sectors leads to cycles that eventually damp out, leading to a stable scenario in which polymorphisms remain in the three sectors without the full accomplishment of the paradigm shift. In all cases, the dynamical effect of the interdependencies between the three sectors induced by the payoff matrix is nicely captured, using the population dynamics approach that we employ here [29–31].

The scenarios just described are the result of numerical simulations of the three-population evolutionary dynamics, for specific sets of parameters (see Methods for details). The question, however, is whether it is possible for us to anticipate and understand (without resorting to simulations) what are the possible scenarios that the model encompasses, and under which circumstances are such scenarios achievable. This is precisely what we address in the following.

**Figure 2.**
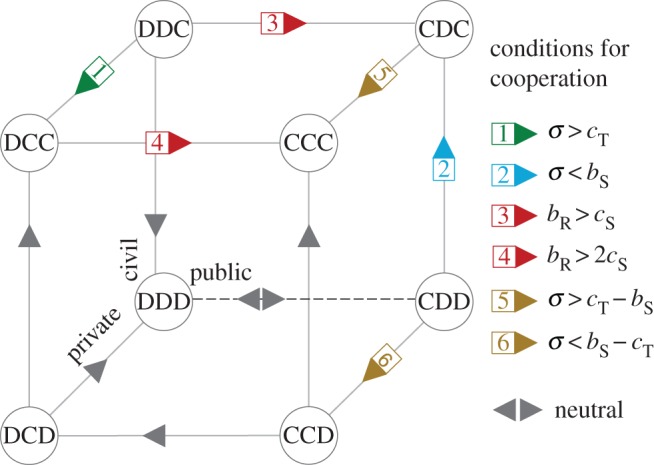
Evolutionary dynamics along the edges of the simplex. When the populations are constrained to move along the edges of the simplex, one is able to derive, analytically, the conditions under which the fixation probability of a cooperator is higher than that of a defector (a condition analogous to risk-dominance of cooperation—see Methods for details). These are indicated along each edge of the simplex, and explicitly written on the legend. The bidirectional arrow represents the neutral evolution associated with the transition of the public sector from the *status quo* into a new paradigm. Dark-grey arrows indicate the direction of evolution along the edges in which the corresponding dynamics is one of unconditional dominance, i.e. the preferred vertex is always the same provided that a set of likely conditions are verified, namely *σ*, *b*_S_, *c*_T_, *c*_S_ > 0. Evolution becomes neutral whenever the mentioned parameters become zero. The numbered arrows indicate the more complex transition scenarios, detailed in the legend. The vertex pointed by the arrow is selected whenever the attached condition is verified. As discussed in the main text, the analysis of these conditions allows us to characterize all possible scenarios anticipated in figure 1 by means of numerical simulations of the evolutionary dynamics of the three populations, encompassing the full cubic space of the simplex represented here.

The behavioural dynamics of the three populations proceeds in a configuration space (or simplex) with the shape of a cube, in which a point of rational coordinates (*x, y, z*), matches a three-population configuration in which a fraction *x* (*y*, *z*) of individuals of the public (private, civil) population cooperate. Consequently, the vertices comprise scenarios in which any of the three populations is either full cooperating (*C*) or full defecting (*D*)—so-called monomorphic configurations—whereas edges represent scenarios in which only one of the populations may exhibit a polymorphic configuration, as illustrated in figure 2.

Owing to the finite size of each population and the stochastic nature of the social-learning process (see Methods), there is always a probability of dynamically reaching a vertex in the configuration space (where one strategy in each population becomes extinct). This means that the population will remain in that configuration until a ‘mutation’ occurs, reflecting the random adoption, by one individual of a population, of another strategy. In the Methods section, we derive analytically the conditions that govern the dynamics along the edges of the cube, associated with scenarios in which one such ‘mutation’ occurs and the dynamics proceeds through social learning until a new vertex is reached. This approximation, so-called *small mutation limit* [38,39], proves insightful in grasping the possible scenarios predicted by a model of this kind. Indeed, as will become clear in the following, this analytical approach allows us to anticipate the same qualitative scenarios shown in figure 1, obtained by means of numerical simulations.

Figure 2 summarizes the conditions that govern the dynamics along the edges of the simplex. The rationale for these analytical conditions is explained in detail in the Methods section. Given the structure of the model, the evolutionary dynamics is generally characterized by pure dominance, whose evolutionary direction relies on the aforementioned conditions. More elaborate models, such as the one discussed in the electronic supplementary material, may include entries in the payoff matrix that lead to more complex scenarios, encompassing either coordination or coexistence. These cases are dealt with exactly in the same way as discussed here.

Because we are interested in how cooperation can emerge and prevail in all three populations, our analysis starts at the point of full defection, that is, vertex DDD, investigating the conditions under which vertex CCC may (or may not) be reached. Out of the three vertices emerging from DDD, the neutral edge DDD–CDD is clearly the most viable, in contrast with the dynamics along the edges DDD–DDC and DDD–DCD: in both cases, cooperators are always worse-off than defectors. Thus, the natural first step towards a paradigm shift comes precisely from the public sector. Neutral drift, in turn, given (i) the population size, (ii) the social environment in which the decision process is embedded, and (iii) the political swaying that characterizes the public sector, is not difficult to envisage as a viable means to traverse the DDD–CDD edge.

Once at the CDD vertex, the most likely edges to be traversed by the populations are the CDD–CDC, (involving a switch of the civil sector) and CDD–CCD (involving a switch of the private sector). The first transition may occur provided the subsidy benefits acquired by the civil sector outweigh the synergistic gains that will be lost if that switch is accomplished. The second transition, on the other hand, may occur provided the difference between the public subsidy and the cost of technological investments outweighs the synergistic gains that will be lost once the private and civil sectors become misaligned.

In general, the likelihood of each path will be determined by the parameters' values in each specific case under study. In this case, as long as *c*_T_ > 0, the path CDD–CDC will always be preferred compared with path CDD–CCD. This result is fully corroborated by the numerical scenarios portrayed in figure 1, involving the full simplex instead of limiting the analysis to the ‘edge-dynamics' that we are able to deal with analytically.

Once in configuration CDC, and as long as the reputational benefits stemming from the civil sector (*b*_R_) outweigh the subsidy costs supported (*c*_S_), the public sector will remain stable in cooperation. In turn, edge CDC–CCC will probably be traversed as the private sector adopts *C*, whenever the advantages of synergy with civil cooperators (*σ*) and the earned subsidies (*b*_S_) overcome the technological costs (*c*_T_). This sequential order of transitions stemming from our analysis of the ‘edge-dynamics’ agrees qualitatively with the full dynamics depicted in figure 1 although, clearly, the shift in the private sector takes place right after the start of the transition of the civil sector.

The new configuration CCC will remain stable provided the reputation benefit (*b*_R_) is greater than twice the cost of subsidies (see condition 4 in figure 2 and figure 1*a*). Otherwise, the path CCC–DCC will ensue (figure 1*b*). Yet, it is noteworthy that this condition is not particularly relevant since, once in the DCC configuration, and provided the stability granted by *σ* < *c*_T_, the alignment between the private and civil sectors will be enough to secure a stable new paradigm, even if the public sector remains misaligned (figure 1*b*). In other words, the achievement of cooperation among all sectors may render the position of the public sector irrelevant, as the private and civil sector will remain coordinated, even if the public sectors turns into defection. Finally, a similar analysis of the balance between the different parameters associated with figure 1*c* is associated with the ‘edge-cycle’ DDD–CDD–CDC–DDC–DDD. Here, the damped oscillations are due to the joint effect of slight mutations (detailed in Methods) and edge conditions 1, 5 and 6 (figure 2), which, respectively, allow and stimulate the incremental departure from the cube face DDD–CDD–CDC–DDC towards the interior of the cube.

Figure 1 demonstrates how the intuitive predictions one establishes based on the analytical estimates displayed in figure 2 are nicely reproduced whenever one performs numerical simulations incorporating the full simplex, instead of analysing the dynamics solely along its edges. Indeed, while the intuition from the analytical estimates remains impressively useful, opening the evolutionary dynamics to the interior of the simplex adds a stabilizing factor, which contributes to damp the cyclic behaviour predicted to the scenario displayed in figure 1*c* leading ultimately to polymorphic configurations.

**Figure 3.**
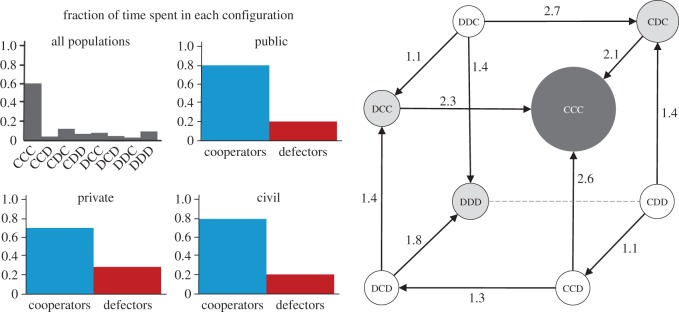
Stationary distributions. The time-series considered in figure 1*a* is a specific initial value solution of the more general dynamical process summarized in figure 2. Here, we portray the relative time spent in each monomorphic state, for the same parameters used in figure 1*a*. Left panel: histograms show the time spent in states where public, private or civil sectors support (cooperators) or avoid (defectors) the paradigm shift. Right panel: here we redraw the simplex from figure 2 with each monomorphic state illustrated with size and colour values that translate the fraction of time spent there (dark and larger means more time). We represent the transitions between states along the edges of the simplex by arrows, whenever the corresponding probability is higher than 1/*Z*. The arrow labels indicate that probability, normalized by 1/*Z*. The dashed line indicates a neutral transition between configurations. Other parameters: *c*_S_ = *c*_T_ = 0.15, *b*_S_ = 0.4, *b*_R_ = 0.8, *σ* = 0.2; (*β* = 0.08, *Z* = 50, see Methods).

An impressive account of the analytical predictions of this approximation can be obtained by computing the stationary distribution (see Methods), which provides information on the fraction of time that the three-populations spend in each vertex of the simplex. In figure 3, we show results for the specific set of parameters corresponding to the scenario portrayed in figure 1*a*. The analysis puts in evidence, once again, the pivotal role played by the civil sector, as its strength will ultimately determine the stability of vertex (CCC). A weaker civil sector, not capable of forming alliances (synergies), will indirectly steer the cost of subsidizing to a disadvantageous stance for public cooperators, eventually leading them into full defection. Furthermore, the feedback of the civil sector into the public sector, by ensuring a sizeable benefit stemming from reputation, is crucial in stabilizing all sectors into cooperation.

## 4. Discussion

The interaction between different individuals within a society, often with conflicting interests, goals and capacities for adaptation, leads to global consequences which are inherently hard to predict and control [21]. In the present case, the intricate and nonlinear interdependencies that accrue to the three main sectors of modern societies make it difficult to systematically analyse their co-evolving dynamics. To apprehend these dynamics is, however, crucial to cope with the complex ecology of decisions in a multi-sector setting. In this quest, a multi-sector analysis provides the lens to regard societies at different scales, with advantages and difficulties analogous to those stemming from the modelling of multi-species ecosystems [22]. In this work, we use at profit those similarities, showing how tools originating in the realm of ecology and population biology may offer new paths and dynamical predictions to the ongoing challenge of understanding social ecosystems associated with both local and global governance. The core model (together with the more specialized model discussed in the electronic supplementary material) allows such an analysis by recasting their interplay in terms of a three-player game of cooperation, involving three populations, one for each sector. To the best of our knowledge, this is the first time such a framework has been developed and employed, naturally stimulated by the problem at stake. In this realm, the core model analysed here introduces a simplified sketch of the interdependencies that exist, often reflecting opposite interests. Our results shed light into the potential key-role that the civil (incumbent) sector may play in rendering feasible a paradigm shift in modern societies. Our findings suggest that public intervention is essential to initiate the change, whereas the synergies that may form between the private and civil sectors prove to constitute important steps in favouring a paradigm shift. The public sector thus acts as an enabler of new (e.g. green) movements that may originate spontaneously within civil society (e.g. civil movements and individuals who voluntarily started to behave greenly, for instance, by separating garbage or starting to use public transportation), but which nevertheless require help regarding consolidation.

Finally, we stress the fact that the role of different sectors within a society and the way they position themselves in their interrelations are culturally dependent. Accordingly, the payoff matrices used to test the proposed framework, both in the core model and in the electronic supplementary material, are tailored to describe relations occurring in a specific cultural arrangement, one that we identify with Western democracies. The motivation for this paper was to analyse the transition from a two-player to a three-player game of political governance and the specific features that leveraged this transition in Western societies. Once again, alternative configurations and positions regarding this transition and its cultural specificities are to be included in new payoff matrices. Afterwards, the overall analysis and methodological procedures follow the very same way.

## 5. Methods

We define three finite populations for each sector of the society; for simplicity, we shall assume that all populations have the same size *Z* *=* *Z*_1_ = *Z*_2_ = *Z*_3_ = 50. In each population, an individual may choose one of two strategies: in favour (*C*, cooperator) or against (*D*, defector) a paradigm shift. Denoting by *xZ* the number of *C*s in the public, *yZ* the number of *C*s in the private and *zZ* the number of *C*s in the civil sectors, the configuration space can be associated with a cube whose vertices correspond to values of (*x,y,z*), where {x,y,z}∈{0,1}, so-called monomorphic configurations. As discussed above, individuals revise their strategies as time goes by, being influenced by the behaviours and successes of others. The success of an individual is fully described by his/her fitness, i.e. the average return obtained from a three-player game defined by the matrix *P_XYZ_* of table 1 (for a more complete version, see electronic supplementary material). Each game involves an individual randomly drawn from each population, leading to a fitness $f_{X\in \{C,D\}}^{}$ given by $f_{X}^{\mathrm{Public}}(x,y,z)=yzP_{XCC}^{\mathrm{Public}}+(1-y)zP_{XDC}^{\mathrm{Public}}+y(1-z)P_{XCD}^{\mathrm{Public}}+(1-y)(1-z)P_{XDD}^{\mathrm{Public}}$, $f_{X}^{\mathrm{Private}}(x,y,z)=xzP_{CXC}^{\mathrm{Private}}+(1-x)zP_{DXC}^{\mathrm{Private}}+x(1-z)P_{CXD}^{\mathrm{Private}}+(1-x)(1-z)P_{DXD}^{\mathrm{Private}}$ and $f_{X}^{\mathrm{Civil}}(x,y,z)=xyP_{CCX}^{\mathrm{Civil}}+(1-x)yP_{DCX}^{\mathrm{Civil}}+x(1-y)P_{CDX}^{\mathrm{Civil}}+(1-x)(1-y)P_{DDX}^{\mathrm{Civil}}$.

Behavioural dynamics is described by a stochastic birth–death process rule [32,40], which describes the social dynamics of *C*s and *D*s in each finite population. At each time step, a randomly selected individual *A* (with fitness *f_A_*) may adopt a different strategy by imitating a randomly chosen individual *B* from the same sector (with fitness *f_B_*) with probability $p={\left[1+e^{-\beta (f_{B}-f_{A})}\right]}^{-1},$ where *β* controls the stochasticity of the imitation process. Additionally, with a probability *μ*—which may be seen as a mutation—individuals explore the strategy space by adopting a randomly chosen strategy.

While a complete characterization of such an evolutionary process is unfeasible for arbitrary *Z* (given the large number of discrete states of the system), the prevalence of each strategy in each population can be readily approximated when *μ* → 0, i.e. in the limit in which mutations are rare [38,39], yet with potential to spark dramatic changes [41] and/or provide the needed instability to fundamentally impact long-term trends in ecosystems [42]. Therefore, here we extend this powerful approach—whose validity has been shown to extend well beyond available analytical estimates [43–45]—to multi-population models. Dynamics is approximated by means of an embedded Markov chain whose states correspond to the eight monomorphic states in which all individuals of each sector share the same strategy. From each monomorphic state, there are three possible transitions, each representing a strategic mutation within one of the three sectors and the following population-wide shift (for an intuitive representation, see figure 2). The respective transition matrix *Λ*, with $\Lambda_{ii}=\rho_{ij}/3(j\neq i)$ and $\Lambda_{ii}=1-\Sigma_{k\neq i}\Lambda_{ik},$ collects the different fixation probabilities *ρ_ij_* that a population at a monomorphic state *i* will end up in another monomorphic state *j*, after one individual in one of its sectors (the mutant or innovator) tries out a different strategy. Following [32,33,46], this probability is given by $\rho_{ij}={\left[1+\Sigma_{l=1}^{Z-1}\Pi_{k=1}^{l}(T_{k}^{-}/T_{k}^{+})\right]}^{-1},$ where $T_{k}^{\pm }=(k/Z)((Z-k)/Z){\left[1+e^{\mp \beta (f_{S_{i}}-f_{Sj})}\right]}^{-1}$ represents the probability that the number *k* of mutants (say, *C*s or *D*s) in a population of *Z – k* residents (*D*s or *C*s, respectively) increases/decreases by one. In figure 2, we represent the conditions that allow the fixation probability of a single *C* (*ρ_C_*) in a population of *Z *– 1 *D*s to be larger than the fixation probability (*ρ_D_*) of a single *D* within *Z* − 1 *C*s, which, in the limit of weak selection [33] and large populations, is equivalent to say that cooperation is risk-dominant. The stationary distribution, *π*, i.e. the fraction of time the population spends in each monomorphic state, is given by the eigenvector of Λ associated with the largest eigenvalue, *πΛ* *=* *π*.

While the limit of rare mutation provides a general picture which can be used to assess the most prevailing configurations (figure 3), one can also extend this analysis to address the most probable trajectories in the interior of the simplex (figure 1). Using the probabilities, $T^{\pm }$, of increasing/decreasing the number of *C*s, it is possible to compute a gradient of selection, $g_{i}(\text{j})=T_{\text{j}}^{+}-T_{\text{j}}^{-}=((Z_{i}-j_{i})/Z_{i})(j_{i}/(Z_{i}-1))\tanh[\beta ({f^{i}}_{C}(\text{j})-{f^{i}}_{D}(\text{j}))/2)]+((Z_{i}-2j_{i})/Z_{i})\mu$ [28,32,40,47] that indicates the most probable direction of evolution in the sector represented by population *i*, given the state of the remaining; **j** identifies a specific configuration (a point in the cube-simplex) of the three populations, i.e. **j **= (*j*_1_, *j*_2_, *j*_3_), where *j_i_* is the number of *C*s in population *i*; *Z*_i_ is the size of population *i*. The fact that we are considering multiple populations calls for the use of a multi-dimensional gradient of selection, in the form ∇(**j**) = {*g*_1_(**j**), *g*_2_(**j**), *g*_3_(**j**)}.The gradient of selection, ∇(**j**) provides information of the most likely direction of evolution of this three-population dynamics [48].

## Supplementary Material

Media summary

## Supplementary Material

Paradigm shifts and the interplay between state, business and civil sectors - Supplementary Material. In the Supplementary Material, we show how the core model proposed in the main text can be extended to treat explicitly the problem of how to shift from a coal based society into a greener (and thus, more sustainable) society.

## Data Availability

Not applicable.
