# Supplementary material for: Paradigm shifts and the interplay between state, business and civil sectors
Source: R Soc Open Sci. 2016 Dec 21;3(12):160753. doi: 10.1098/rsos.160753 (PMC5210695; doi:10.1098/rsos.160753)
Supplement: Media summary [file rsos160753supp1.doc]

**Media summary**

The interaction between individuals within a society, organized in different sectors and with growing emphasis on civil institutions, is inherently complex and difficult to analyze. Here we employ tools from ecology and population biology to comprehend those interrelations. Our results evince the potential key-role that the civil sector has in enabling a paradigm shift in modern societies, especially if supported by the public sector. Interestingly, a later civil-private coordination may exempt that support. This dynamical perspective on inter-sectorial coordination can constitute a key asset for political actors concerned with the complex ecology of decisions that accrue to multi-level governance.
