# Supplementary material for: Paradigm shifts and the interplay between state, business and civil sectors
Source: R Soc Open Sci. 2016 Dec 21;3(12):160753. doi: 10.1098/rsos.160753 (PMC5210695; doi:10.1098/rsos.160753)
Supplement: Paradigm shifts and the interplay between state, business and civil sectors - Supplementary Material. In the Supplementary Material, we show how the core model proposed in the main text can be extended to treat explicitly the problem of how to shift from a coal based society into a greener (and thus [file rsos160753supp2.pdf]

Supplementary Material

Paradigm shifts and the interplay between state, business and civil sectors

Sara Encarnação[a,b,c,d], Fernando P. Santos[d,b], Francisco C. Santos[d,b], Vered Blass[e], Jorge M. Pacheco[f,b], and Juval Portugali[a,§]

(a) ESLab Environmental Simulation Laboratory, Tel Aviv University, Tel Aviv 69978, Israel.

(b) ATP-group, P1649-003 Lisboa Codex, Portugal.

(c) Interdisciplinary Centre of Social Sciences – CICS.NOVA – FCSH/UNL, Avenida de Berna, 26-C, 1069-061 Lisboa, Portugal.

(d) INESC-ID and Instituto Superior Técnico, Universidade de Lisboa, IST-Taguspark, 2744-016 Porto Salvo, Portugal.

(e) Faculty of Management, Tel Aviv University, Tel Aviv 69978, Israel.

(f) Centro de Biologia Molecular e Ambiental and Departamento de Matemática e Aplicações, Universidade do Minho, 4710 - 057 Braga, Portugal.

[§] **Corresponding Author**
Juval Portugali
ESLab (Environmental Simulation Laboratory),
Department of Geography and the Human Environment,
Tel Aviv University, Tel Aviv 69978, Israel.
Phone: +972 (0) 3 640 8661; E-Mail: juval@post.tau.ac.il.

Achieving sustainable societies often requires the intervention of different sectors due to the intricate nature of the problems at hand. Governments, private firms and individuals can all contribute to a problem but can all also have the capacity to contribute to a solution. However, when the solution resides on a change in the *status quo* it is not easy to accommodate a milieu of (conflicting) interests. The specific case of this framework discloses the complex nature of the interdependencies between all three sectors (Public, Private and Civil). Thus, the advancement of methodologies to comprehend the dynamics inherent to this 3-player game reveals itself imperious.

Here we make use of the three populations' core model presented in the main text, to study a more specific case of a paradigm shift towards more sustainable societies, where players can adopt one of two available strategies — simply saying, for (cooperation) and against (defection) "green" policies. By incorporating an additional set of parameters, which may seem closer to contemporary policy instruments, we are able to uncover some non-trivial emerging dynamics assuming the interdependent success of the strategies used in the different populations. Irrespectively of this, our results confirm that, departing from a small fraction of cooperators (that mimics the real and present *status quo*) the conjugated action of the Private and Civil sectors, alone, is not sufficient to achieve a stable state of full cooperation towards the adoption of green actions. To achieve it, Cooperation from the Public sector will be mandatory, even if at a later stage, when cooperation among all populations is achieved, the contribution of the public sector is no longer needed.

Model, parameters and payoff matrix

As before, we assume three different finite populations, each representing one sector of society, namely, the Public, Private and Civil sectors. The Public sector represents government or government institutions, the Private sector represents companies that produce or sell products and the Civil sector is composed by citizens that can manifest (or not) pro-environmental behaviour, individually or by participating in, e.g., NGOs.

Each individual (in any of the three populations) can adopt one of two strategies: in favour of green policies (cooperators - ***C***s) and against it (defectors - ***D***s). The individual payoff earned in any one encounter depends

on the strategy profile given each participant decision. An encounter always involves the participation of three individuals, each belonging to a different population. As a result, each individual will acquire a payoff that reflects the mechanisms at stake, and which is encoded in the so-called payoff matrix (Table S1).

Strategies			Payoffs accruing to each player		
Public	Private	Civil	Public	Private	Civil
C	C	C	$-S$	$S\delta$	$(1-\delta)S$
C	C	D	$-S$	S	0
C	D	C	$\varepsilon\gamma + \Delta 1 - S$	$\varepsilon(1-\gamma) - P_b$	$(z-1)P_b + \Delta 1 + S$
C	D	D	$\varepsilon\gamma$	$\varepsilon(1-\gamma)$	0
D	C	C	$-P$	$\Delta 2$	$(z-1)P + \Delta 2$
D	C	D	0	0	0
D	D	C	$-P$	$\varepsilon - P_b$	$(z-1)(P + P_b)$
D	D	D	0	ε	0

Table S1. Payoff matrix, where C indicates a Cooperator and D a Defector.

This payoff structure asserts that, when encountering both Private and Civil cooperators (***C***s), Public ***C***s can allocate an amount δ of subsidies (*S*) to Private ***C***s and (*1-δ*) to Civil ***C***s [1]. Civil cooperators can engage in a form of costly punishment [2, 3] when encountering defectors, by punishing (by charging a cost *P*) Public ***D***s and boycotting Private ***D***s (by imposing a charge P_b) [4-7]. Both *P* and P_b are frequency dependent and inversely proportional to the number of civil cooperators present in the population, simulating the scale effect of sharing the costs of punishing [8]. Social punishment [2] as a punitive mechanism can induce considerable costs to both Public and Private sectors [6]. One way to overcome this confrontation and resolve or mitigate possible contrasting or even conflicting interests is through green alliances and partnerships between and among sectors. We treat alliances as voluntary approaches that surpass legal obligation [9]. These might include eco-labelling frameworks and corporate social responsibility (CSR), among other mechanisms. They are understood as complementarities that can promote collective action [10] and reduce the environmental impacts of each individual agent [11]. Our assumption is that when “win-win” alliances are established synergistic effects might emerge and enhance cooperation levels.

In our 3-player game, synergies will exist when cooperators of two populations meet a defector of a third population. Thus, Civil and Public cooperators will share synergistic effects (Δ1) when meeting a Private

defector and Civil and Private cooperators will share synergistic effects (Δ2) when encountering Public defectors. We restrict our synergies' types to those shared with the Civil sector alone. This constraint enables us to study how civil empowerment can impact cooperation towards common environmental goals.

Although social punishment can be used as a mechanism to change agents' behaviours, adaptation to new and greener technical and procedural processes can be highly costly to companies. Some Private defectors might not be willing (or capable) to make this adaptation and thus continue to produce and sell "non-green" products, from which they will receive a higher differential profit (ε). Albeit this advantage, Public (Cs) will have the possibility to counteract by imposing green taxes (γ) to ε, and thus reducing the initial market leverage of the former. Null payoffs are incorporated as baselines for comparing between businesses as usual strategies and pro environmental behaviour.

From the above payoff matrix, and due to existing interdependences among State, Business and Society, it is not clear if policies that only aim at the supply side (as green taxes do) will fall short on promoting cooperation towards solving environmental problems, when not integrated in set of mix policy instruments [12]. To clarify this matter further we need to take into account the evolutionary dynamics emerging from the interaction between agents of all three populations, as detailed in the next section.

Population dynamics

As before, the resulting dynamics of the aforementioned model can be accessed through the study of a three-dimensional space that encompasses all possible population configurations, regarding the number of individuals adopting each strategy. Let us assume three populations of size Z and that each configuration is defined by a point (xZ,yZ,zZ), which matches a state in which a fraction x (y, z) individuals of the Public (Private, Civil) population adopt green policies (Cooperates). Thereby, the vertexes comprise the scenarios in which the three populations are either full cooperating (***C***) or full defecting (***D***) (Figure S1).

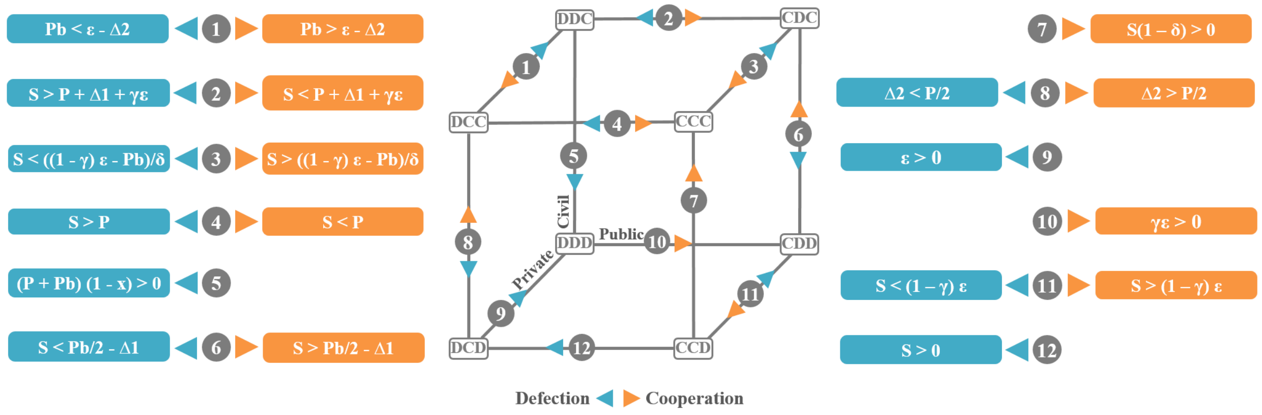

Figure S1.Three player representation cube (Middle). Each edge will be described by conditions that promote the a higher fixation probability of cooperation (orange) or defection (blue), or both (Left and Right).

As in the main text, we derive the conditions that govern the dynamics in the edges of the cube, which stand as a convenient simplification for dynamics over all states (including the inside of the cube) under the assumption of rare mutations [13-15].

Since we are interested in how cooperation can emerge and prevail, let us start at the point of full defection in all sectors (DDD) and we try to uncover the conditions that will lead to coordination of all sectors – vertex (CCC). Concerning the edge conditions that translate into a higher fixation probability of a single cooperator over the fixation probability of a single defector (Figure S1), we observe that in a state of full defection (DDD), the Civil and Private sectors find themselves constrained by conditions that prevent cooperation (edges 5 and 9 in Figure S1). For the Private sector (edge 9), cooperation prevents the extra profit earned by not following pro-environment policies, without the advent of compensation. For the Civil sector (edge 5), cooperation requires the cost of being active in social punishment to both Public and Private defectors. By contrast, cooperation will always increase when Public cooperators impose green taxes (γ) on Private defectors extra profit (ε) for selling or producing non-green products (edge 10). This state-driven intervention might be seen as a way to address market negative externalities (e.g. Pollution) but it is not sufficient to achieve vertex (CCC). Nonetheless, new possibilities arise that could disentangle cooperation levels when the world reaches vertex (CDD), as new instruments become available (e.g. subsidies and synergies). The pathway to full cooperation implies adopting one of two possible routes, i.e., through Civil engagement in cooperation (edge 6) or Private engagement (edge 11).

The distinction between these two routes is central as it defines two different policy philosophies. By first engaging with the Civil sector (edge 6), subsidies given by Public cooperators will be used to mitigate the cost that Civil cooperators must endure while boycotting Private defectors. At the same time, the financial effort that Public cooperators must bear can be balanced with the existence of Public-Civil synergies. On the other hand, if conditions that enable Civil cooperation are not met, then Private engagement in cooperation might solve the puzzle (edge 11). Here, cooperation will be solely dependent on the balance between subsidies gain and taxes paid by Private agents. The amount of subsidies given will have to cover the extra profit (after deducing the environmental taxes). However, if the Public sector is not able to meet this criterion, as subsidies are costly, then Private agents will have more to gain if they maintain their strategy (Ds), and cooperation will thereby perish.

The analysis above becomes clear when calculating the stationary distribution (see Methods) using a set of parameters' values that will promote cooperation in all possible conditions (Figure S2). Route DDD→CDD→CDC→CCC (through Civil engagement first) is the most advantageous the majority of the times.

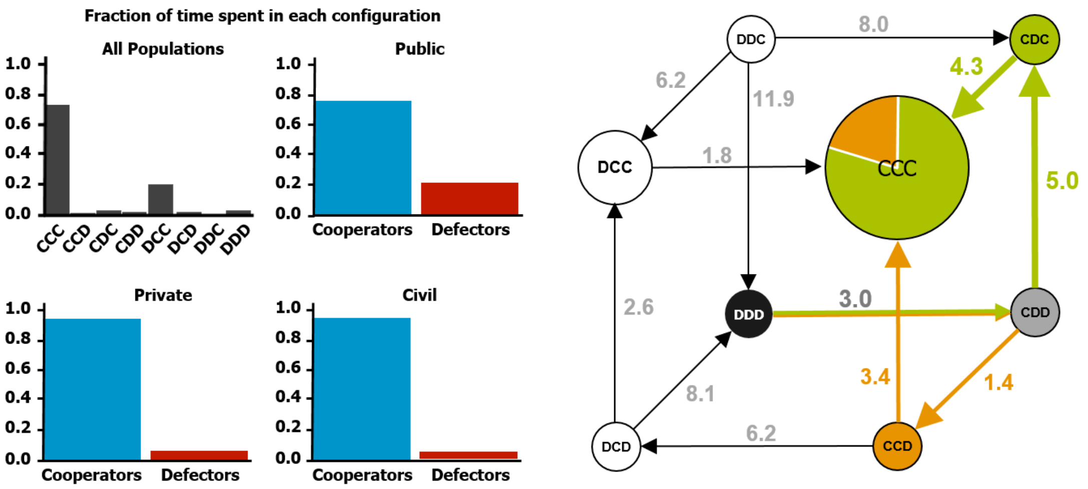

Figure S2. Stationary distribution when all possible conditions promote cooperation (P=0.18; P_b=0.16; Δ1=0.1; Δ2=0.19; ε=0.2; Υ=0.33; Ѕ=0.15; δ=0.5). All sectors spend most of the time as cooperators, and the world will remain 70% of the time in configuration CCC (Left panel). Routes DDD->CDD->CDC->CCC and DDD->CDD->CCD->CCC are the most probable to reach state CCC. The relative weight of transitions between monomorphic states is represented, both along edges and within the states, by two colours (orange: DDD->CDD->CCD->CCC and green: DDD->CDD->CDC->CCC). Edge numbers give the ratio between the actual transition probability against neutral drift probability – 1/Z.

These results seem to suggest the need to reinforce Civil empowerment, whether through direct action of NGOs and/or through Public steering, by establishing conditions to strengthen citizens awareness and pro-environmental behaviour, as well as increasing participation rates on policies design. Empirical evidence concur to these findings in that it has been shown that NGOs ability to influence the outcome of international environmental negotiations has been limited, although some of their strategies as outsiders (such as mass demonstrations and high media cover) have been capable of achieving successful results [16, 17].

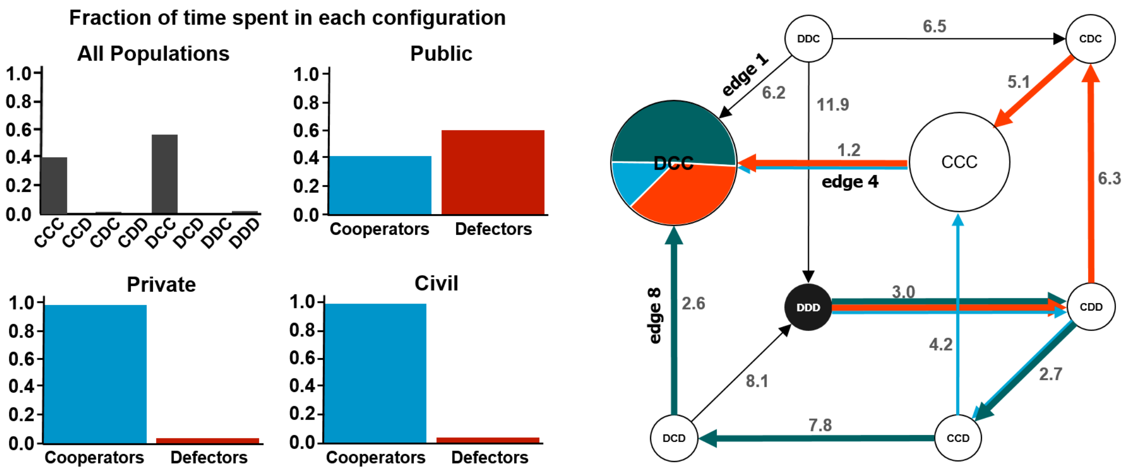

Figure S3. Stationary distribution when vertex (CCC) is unstable by changing edge 4 condition (P=0.18; P_b=0.16; Δ1=0.1; Δ2=0.19; ε=0.2; ϒ=0.33; S=0.19; δ=0.5). The world will spend most of the time in state DCC, followed by state CCC. Private and Civil sectors will remain highly cooperative though the Public sector will adopt defection more often (Left panel). The relative weight of transitions between monomorphic states is represented, both along edges and within the states, by two colours.

The strength of the Civil sector will determine the stability of vertex (CCC). A weaker Civil sector, not capable of punishing effectively or forming alliances (synergies), will indirectly steer the cost of subsidizing to a disadvantageous balance for Public cooperators and eventually lead them to full defection. The significance of a stronger Civil sector becomes even more prominent when we analyse the case where vertex (CCC) becomes unstable (Figure S3). Here, in edge 4, cooperation is not promoted anymore (since $S>P$ – see Figure S1) and the Public sector will change to full defection.

In the above case, a strong Civil sector is needed to not only commit to social punishment but also to alliances with the Private sector (edges 1 and 8). When this is achieved the world will spent most of the time in state DCC, where the Public sector does not participate, but the demand and supply sides (Private and Civil) keep high levels of cooperation towards pro-environmental behaviours.

Our findings suggest that *i)* high values of synergy between Civil and Private sectors and *ii)* the existence of efficient mechanisms of punishment, both contribute to reduce the importance of the Public Sector (Government) in maintaining overall cooperation.

This model is significantly more complex than the core model introduced in the main text. Naturally, it still does not account for all the circumstances that underlie the relations between all three sectors in reality. Unbalanced power relations and conflicting interests can substantially undermine governance processes [10, 17, 18]. Nonetheless, the aforementioned idiosyncrasies can be address following the same methodological procedure that already led us from the core model (main text) to the one presented here (**SI**). Other extensions might also incorporate more heterogeneous populations that could account for social and economic differentiation and further test how unbalanced power relations can in effect change social game dynamics in specific cases, such as green alliances.

The intricate role of the Public sector in empowering Civil society is mirrored in the claims of empirical research on the need to increase independence and strength of NGOs, by guaranteeing that symbolic alliances with no real and substantive outputs are not the majority [19-22]. It should also aim at regulating the information that is accessible to consumers [3] and thus diminish suspicions and raise confidence in engaging in pro-environmental behaviours [20].

Boycotting is an expression of political consumerism that can empower civil society, however it relies on highly informed and active citizens in order to reach its full potential [12]. Public policies in this domain should thus serve as the mainstay for heightening cooperation. How this is achieve can also be dependent on the institutional environment of each country [23]. And although this conundrum is beyond the scope of this paper, we acknowledge evidences that show that the effectiveness of alliances between non-state actors (e.g. NGOs and companies) and their impact on public policies can be highly marked by the institutional context in which they reside [1, 21, 24].

Centrally-driven regulations, together with "soft" steering mechanisms, can contribute to increase levels of awareness and change perceptions and, more importantly, behaviours – the backbones for the evolution of

social norms [25-27]. The message is clear here: a cooperative society towards sustainable worlds will only work with the involvement of all sectors of society.

References

1 Gutowski, T., Murphy, C., Allen, D., Bauer, D., Bras, B., Piwonka, T., Sheng, P., Sutherland, J., Thurston, D., Wolff, E. 2005 Environmentally benign manufacturing: Observations from Japan, Europe and the United States. *J Clean Prod*. **13**, 1-17. (doi: 10.1016/j.jclepro.2003.10.004)

2 Balabanis, G. 2013 Surrogate Boycotts against Multinational Corporations: Consumers' Choice of Boycott Targets. *British Journal of Management*. **24**, 515-531. (doi: 10.1111/j.1467-8551.2012.00822.x)

3 Delacote, P., Montagné-Huck, C. 2012 Political consumerism and public policy: Good complements against market failures? *Ecological Economics*. **73**, 188-193. (doi: 10.1016/j.ecolecon.2011.10.020)

4 Albrecht, C.-M., Campbell, C., Heinrich, D., Lammel, M. 2013 Exploring why consumers engage in boycotts: toward a unified model. *Journal of Public Affairs*. **13**, 180-189. (doi: 10.1002/pa.1473)

5 Copeland, L. 2014 Conceptualizing Political Consumerism: How Citizenship Norms Differentiate Boycotting from Buycotting. *Political Studies*. **62**, 172-186. (doi: 10.1111/1467-9248.12067)

6 Franks, D. M., Davis, R., Bebbington, A. J., Ali, S. H., Kemp, D., Scurrah, M. 2014 Conflict translates environmental and social risk into business costs. *Proc Natl Acad Sci USA*. **111**, 7576-7581. (doi: 10.1073/pnas.1405135111)

7 Stolle, D., Hooghe, M., Micheletti, M. 2005 Politics in the supermarket: Political consumerism as a form of political participation. *Int Polit Sci Rev*. **26**, 245-269. (doi: 10.1177/0192512105053784)

8 Boyd, R., Gintis, H., Bowles, S. 2010 Coordinated Punishment of Defectors Sustains Cooperation and Can Proliferate When Rare. *Science*. **328**, 617-620. (doi: 10.1126/science.1183665)

9 OECD. 2000 *Voluntary Approaches for Environmental Policy: An Assessment*. OECD Publishing.

10 Evans, P. 1996 Government action, social capital and development: Reviewing the evidence on synergy. *World Development*. **24**, 1119-1132. (doi: 10.1016/0305-750X(96)00021-6)

11 Albino, V., Dangelico, R. M., Pontrandolfo, P. 2012 Do inter-organizational collaborations enhance a firm's environmental performance? a study of the largest U.S. companies. *Journal of Cleaner Production*. **37**, 304-315. (doi: 10.1016/j.jclepro.2012.07.033)

12 Daugbjerg, C., Sønderskov, K. M. 2012 Environmental Policy Performance Revisited: Designing Effective Policies for Green Markets. *Political Studies*. **60**, 399-418. (doi: 10.1111/j.1467-9248.2011.00910.x)

13 Fudenberg, D., Imhof, L. A. 2006 Imitation processes with small mutations. *J Econ Theory*. **131**, 251-262. (doi: 10.1016/j.jet.2005.04.006)

14 Imhof, L. A., Fudenberg, D., Nowak, M. A. 2005 Evolutionary cycles of cooperation and defection. *Proc Natl Acad Sci USA*. **102**, 10797-10800. (doi: 10.1073/pnas.0502589102)

15 Santos, F. C., Pacheco, J. M., Skyrms, B. 2011 Co-evolution of pre-play signaling and cooperation. *Journal of Theoretical Biology*. **274**, 30-35. (doi: 10.1016/j.jtbi.2011.01.004)

16 Arts, B. 2002 Green alliances? of business and NGOs. New styles of self-regulation or ?dead-end roads?? *Corporate Social Responsibility and Environmental Management*. **9**, 26-36. (doi: 10.1002/csr.3)

17 Rietig, K. 2011 Public pressure versus lobbying - how do Environmental NGOs matter most in climate negotiations? Working Paper: Centre for Climate Change Economics and Policy and Grantham Research Istitute on Climate Change and the Environment; December, 2011. Report No.: 79 and 70.

18 Jordan, A., Wurzel, R. K., Zito, A. 2005 The rise of 'new'policy instruments in comparative perspective: has governance eclipsed government? *Political studies*. **53**, 477–496. (doi: 10.1111/j.1467-9248.2005.00540.x)

19 Baur, D., Schmitz, H. P. 2012 Corporations and NGOs: When Accountability Leads to Co-optation. *Journal of Business Ethics*. **106**, 9-21. (doi: 10.1007/s10551-011-1057-9)

20 Delmas, M. A., Burbano, V. C. 2011 The drivers of greenwashing. *California Management Review*. **54**, 64-87. (doi: 10.1525/cmr.2011.54.1.64)

21 Doh, J. P., Guay, T. R. 2006 Corporate Social Responsibility, Public Policy, and NGO Activism in Europe and the United States: An Institutional-Stakeholder Perspective. *Journal of Management Studies*. **43**, 47–73. (doi: 10.1111/j.1467-6486.2006.00582.x)

22 Jamali, D., Keshishian, T. 2009 Uneasy Alliances: Lessons Learned from Partnerships Between Businesses and NGOs in the context of CSR. *Journal of Business Ethics*. **84**, 277-295. (doi: 10.1007/s10551-008-9708-1)

23 Klijn, E.-H. 2008 Governance and Governance Networks in Europe: An assessment of ten years of research on the theme. *Public Management Review*. **10**, 505-525. (doi: 10.1080/14719030802263954)

24 Arts, B., Mack, S. 2003 Environmental NGOs and the Biosafety Protocol: a case study on political influence. *European Environment*. **13**, 19-33. (doi: 10.1002/eet.309)

25 Kinzig, A. P., Ehrlich, P. R., Alston, L. J., Arrow, K., Barrett, S., Buchman, T. G., Daily, G. C., Levin, B., Levin, S., Oppenheimer, M., *et al.* 2013 Social Norms and Global Environmental Challenges: The Complex Interaction of Behaviors, Values, and Policy. *BioScience*. **63**, 164-175. (doi: 10.1525/bio.2013.63.3.5)

26 Kollmuss, A., Agyeman, J. 2002 Mind the Gap: Why do people act environmentally and what are the barriers to pro-environmental behavior? *Environmental Education Research*. **8**, 239-260. (doi: 10.1080/13504620220145401)

27 Peattie, K. 2010 Green Consumption: Behavior and Norms. *Annual Review of Environment and Resources*. **35**, 195-228. (doi: 10.1146/annurev-environ-032609-094328)
